# Suppressed tumorigenicity of human endometrial cancer cells by the restored expression of the DCC gene

**DOI:** 10.1054/bjoc.1999.0943

**Published:** 2000-01-18

**Authors:** H Kato, Y Zhou, K Asanoma, H Kondo, Y Yoshikawa, K Watanabe, T Matsuda, N Wake, J C Barrett

**Affiliations:** Department of Reproductive Physiology and Endocrinology and Department of Pathology, Medical Institute of Bioregulation, Kyushu University, Tsurumihara 4546, Beppu City, Oita, 874- 0838, Japan; Laboratory of Molecular Carcinogenesis, National Institute of Environmental Health Sciences, National Institute of Health, PO Box 12233, Research Triangle Park, NC, 27709, USA

**Keywords:** tumour suppression, tumour suppressor gene, DCC, endometrial carcinoma, apoptosis

## Abstract

To obtain functional evidence for DCC as a tumour suppressor associated with endometrial cancer, the human DCC cDNA encoding a complete open reading frame (ORF) was transfected into highly tumorigenic human endometrial carcinoma cells, HHUA and Ishikawa in which DCC expression was completely deleted. Reconstituted expression of DCC in HHUA had little effect on in vitro growth, but suppressed tumour formation in mice completely. The clones from Ishikawa had abundant DCC expression similar to that in normal endometrium. Their growth in vitro was suppressed and showed apoptotic phenotype. Lower levels of DCC expression in the prolonged passaged clones did not induce apoptosis, but still had the potential to suppress tumorigenicity. These observations imply a role of DCC in regulation of normal endometrial cell growth, and categorize DCC as the tumour suppressor gene for endometrial cancer. © 2000 Cancer Research Campaign


British Journal of Cancer (2000) **82**(2), 459–466
© 2000 Cancer Research Campaign
Article no. bjoc.1999.0943

### Suppressed tumorigenicity of human endometrial cancer cells by the restored expression of the DCC gene

**H Kato[1], Y Zhou[1], K Asanoma[1], H Kondo[1], Y Yoshikawa[2], K Watanabe[1], T Matsuda[1], N Wake[1] and JC Barrett[3]**

[1]Department of Reproductive Physiology and Endocrinology and [2]Department of Pathology, Medical Institute of Bioregulation, Kyushu University, Tsurumihara 4546, Beppu City, Oita 874- 0838, Japan; [3]Laboratory of Molecular Carcinogenesis, National Institute of Environmental Health Sciences, National Institute of Health, PO Box 12233, Research Triangle Park, NC 27709, USA

**Summary** To obtain functional evidence for DCC as a tumour suppressor associated with endometrial cancer, the human DCC cDNA encoding a complete open reading frame (ORF) was transfected into highly tumorigenic human endometrial carcinoma cells, HHUA and Ishikawa in which DCC expression was completely deleted. Reconstituted expression of DCC in HHUA had little effect on in vitro growth, but suppressed tumour formation in mice completely. The clones from Ishikawa had abundant DCC expression similar to that in normal endometrium. Their growth in vitro was suppressed and showed apoptotic phenotype. Lower levels of DCC expression in the prolonged passaged clones did not induce apoptosis, but still had the potential to suppress tumorigenicity. These observations imply a role of DCC in regulation of normal endometrial cell growth, and categorize DCC as the tumour suppressor gene for endometrial cancer. © 2000 Cancer Research Campaign

**Keywords:** tumour suppression; tumour suppressor gene; DCC; endometrial carcinoma; apoptosis

Endometrial carcinoma is one of the most common gynaecological malignancies. Despite the high morbidity associated with this neoplasia, little is known about its underlying genetic mechanisms. However, recent investigations have documented the involvement of a few oncogenes and tumour suppressor genes in endometrial carcinoma. Among them a high incidence of allelic loss has been noted for chromosome 18q in this tumour (Imamura et al, 1992). The region within or near the chromosome region 18q21, which is involved in deletions in endometrial cancers, contains the *DCC* gene. Loss of appropriate transcription in most endometrial carcinoma cell lines and surgically resected tumours (Gima et al, 1994; Enomoto et al, 1995; Ronnett et al, 1997) is compatible with the assumption that inactivation of the *DCC* gene has a role in endometrial carcinoma development. Similarly, significant incidence of loss of heterozygosity (LOH) on chromosome 18q and decreased or absent expression of the *DCC* gene comparing with the normal counterparts were revealed in a variety of other human malignancies of epithelial origin as well as gastric, pancreatic, breast and prostate cancers (Devileei et al, 1991; Neuman et al, 1991; Hoene et al, 1992; Gao et al, 1993; Miyake et al, 1993; Scheek et al, 1993; Brewster et al, 1994). However, there is little proof to categorize *DCC* directly as a tumour suppressor gene. Localized somatic mutations predicted to inactivate the remaining DCC allele have been identified only in a fraction of colorectal cancers (Cho et al, 1994). The length and complexity of the *DCC* gene restrict the demonstration of *DCC* involvement in various malignancies including endometrial carcinomas. A single chromosome 18 transfer into endometrial carcinoma cells has been shown to suppress tumorigenicity, whereas growth properties in vitro were not significantly affected (Yamada et al, 1995). However, it remains unknown whether the suppression of tumour formation was due to *DCC* expression or to expression of some other genes on the transferred chromosome. The recent data define a minimally lost region on chromosome 18q21 in a few carcinomas, which contains at least two candidate tumour suppressor genes, *DPC4* and *DCC* (Thiagtlingam et al, 1996). The deletion analysis further suggested genetic heterogeneity in colorectal carcinomas, with *DPC4* the deletion target of chromosome 18q21 in up to a third of the cases, and *DCC* or neighbouring gene the target in the remaining tumours. However, retained alleles of *DPC4* are mutated in only a fraction of colorectal cancers that suffers 18q LOH (Kei et al, 1996; Takagi et al, 1996).

DCC encodes for a membrane-bound protein with immunoglobulin like and fibronectin type III domains and a unique cytoplasmic domain (Fearon et al, 1990). The first two domains are characteristic of an adhesion molecule belonging to the immunoglobulin superfamily, the members of which are expressed mainly on cells of the nervous and immune systems. These structures of DCC provide the possibility that DCC associates with cell differentiation as an adhesion molecule. Localization of DCC protein in a subset of differentiated cells would support this (Hedrick et al, 1994). Recently, DCC protein was found to be a Netrin-1 receptor in neural cells (Keino-Masu et al, 1996). Netrin-1 is related to the extracellular matrix protein laminin and secreted by floorplate cells as a chemoattractant for commissural axons in the vertebrate spinal cord (Kennedy et al, 1994). Netrin-1 or DCC-deficient transgenic mice showed the identical phenotype, which exhibits the defects in spinal commissural axon projections and in brain morphogenesis (Serafini et al, 1996; Fazel et al, 1997). Thus, Netrin-1–DCC signalling plays a crucial role in nervous system development. Although there are few data to define the role of DCC in non-neural cells, Mehlen et al (1998) found that DCC, which was a caspase substrate, could induce apoptosis in the

*Received 24 February 1999*
*Revised 27 July 1999*
*Accepted 5 August 1999*

*Correspondence to:* H Kato absence of ligand binding, but block apoptosis when engaged by netrin-1 in human embryonic kidney and colonic carcinoma cells. The data suggest that DCC functions as a tumour suppressor protein by inducing apoptosis in settings in which ligand is unavailable. Klingelhutz et al (1995) also demonstrated a direct role of DCC in tumour suppression of nitrosomethylurea-transformed tumorigenic human papillomavirus (HPV)-immortalized human epithelial cells (1811-NMU-T1 cells). This observation supports the function of DCC as a tumour suppressor. However, DCC-deficient transgenic mice showed no increased incidence of colorectal cancers (Fazel et al, 1997). Therefore, the role of DCC in suppression of the malignant phenotype still remains to be elucidated.

Reconstituted DCC expression in the DCC-negative carcinoma cells is able to provide a clue to its functions. In the present study, we investigated the expression levels of DCC protein in human endometrium to define the association with altered DCC expression and endometrial carcinoma development. Then, we examined the effect of reconstitution of DCC expression on the malignant phenotypes exhibited by endometrial carcinoma cells. Reconstituted levels of DCC protein in the DCC-non-expressing carcinoma cells Ishikawa were almost analogous to the level detected physiologically in human endometrium. We now show here that the wild-type full-length DCC cDNA can inhibit both in vitro growth and tumorigenicity in Ishikawa endometrial carcinoma cells, and can inhibit tumorigenicity in HHUA endometrial carcinoma cells. A potential of DCC to induce apoptotic cell death at least partly contributes to the inhibitory effects. Moreover, tumorigenic revertants originated from the non-tumorigenic transfectants abrogate the exogenous DCC cDNA expression.

### MATERIALS AND METHODS

### Cell lines and plasmid transfection

Endometrial carcinoma cell lines (HHUA and Ishikawa) were propagated in high glucose Dulbecco's Modified Eagle's medium (Gibco-BRL-Life Technologies) supplemented with 10% fetal bovine serum (FBS; Medical Link, Yokohama, Japan). All cell lines were negative for the presence of *Mycoplasma*. HHUA and Ishikawa cells expressing full-length DCC were obtained by transfection with the mammalian expression vector pDCCCMV-S. The cells were transfected with a mixture of 5 μg plasmid DNA and cationic liposomes [Lipofectin reagent (Gibco-BRL-Life Technologies)]. Approximately 48 h after transfection, selection in G418 at 600 μg $ml^{-1}$ was started. Single G418-resistant clones were trypsinized and established as clonal cell lines. The cell lines were maintained in G418 at 400 μg $ml^{-1}$.

### RT-PCR analysis of DCC gene expression

Total RNA was prepared from the cell lines, clones and surgically-removed normal human uterine endometrium using the acid guanidinium thiocyanate method (Chomoczynski et al, 1987). Three micrograms of the total RNAs were reverse transcribed (RT) into first-strand cDNA with random hexamer and the Gene Amp, RNA polymerase chain reaction (PCR) core kit (Perkin-Elmer, NJ, USA). The cDNA was then amplified by PCR using primer pairs derived from the transfected DCC cDNA sequence. Four sets of primers covering the whole sequence of DCC were used to analyse the expression. Primer 1 for exons 1–7, sense; 5′-ATGGAGAATAGTCTTAGATG-3 antisense; 5′-CAATGAGCTGTGCACTGGAC-3, primer 2 for exons 7–16, sense; 5′-ATGAGGCTGGAAATGCCCAG-3′ antisense; 5′-ATAGACCTGGTGGTGGCACT-3′, primer 3 for exons 16–24 sense; 5′-TGCCGGAGAAGGAGTTCCTC-3′, antisense; 5′-GCTTCCCAGTTGGGTTTCTG-3′ and primer 4 for exons 24–29, sense; 5′-ACCAGTCAGCCACAGCCAGT-3 antisense; 5′-CTTGGACAGGTGTGCTGT-3 were synthesized for the inter-exon PCR. PCR condition included denaturation for 1 min at 94°C, annealing for 1 min at 56–60°C and extension for 2 min at 72°C. RT-PCR products were electrophoresed on 1.2% agarose gels and transferred to Nylon membrane (Hybond-N+, Amersham). Their identities were confirmed by hybridization with a $^{32}P$-labelled DCC cDNA probe purified from pDCCCMV-S.

### Immunohistochemical and Western blot analyses of DCC expression

Surgically-resected normal human endometrium was fixed in 10% formalin and sections were made onto glass slides. These slides were reacted with antibodies recognizing intracellular DCC domain or extracellular domain (15041A and 15031 respectively; Pharmingen, San Diego, CA, USA), according to the manufacturer's recommendations. After the incubation of the primary antibody, signals were detected with the anti-mouse biotin-labelled antibody following the treatment with avidin-peroxidase and DAB.

Cell lines or tissue homogenates were solubulized in Tris-buffered saline (25 mM Tris (hydroxymethylaminomethane), pH 8) with detergents (1% Nonidet P-40, and 0.1% sodium dodecyl sulphate (SDS)) and protease inhibitors (5 μg $ml^{-1}$ aprotinin, 2 μg $ml^{-1}$ leupeptin, 100 μg $ml^{-1}$ phenylmethylsulphonyl fluoride, and 1 mM EDTA). Protein concentrations were then determined. Unprocessed cell line/tissue lysates underwent SDS polyacrylamide gel electropheresis (SDS-PAGE) (100 μg per lane) and transblot semi-dry transfer (Bio-Rad, Hercules, CA, USA) to nitrocellulose membranes (Schleicher and Schuell, Germany). The primary DCC antibody (Ab-1) (Oncogene Science, NY, USA) was used at 1 μg $ml^{-1}$, and the secondary goat–anti-mouse antisera coupled to horseradish peroxidase (Amersham) was used at a 1:10 000 dilution. The antigen–-antibody complex was detected by enhanced chemiluminescence (ECL; Amersham) and subsequent exposure to Hyperfilm (Amersham).

### Evaluation of in vitro transformed phenotype and tumorigenicity

The greatest cell number after reaching confluency was counted to calculate the saturation density. For the evaluation of anchorage independent growth, a single cell suspension ($10^3$) of parents and clones in 0.3% agar were overlaid on 0.5% basal agar. Colony-forming efficiencies, based on colonies with a diameter of > 0.2 μm, were scored after 4 weeks' observation. To evaluate tumorigenicity in vivo, $5 \times 10^6$ cells in 0.2 ml of serum-free media were inoculated subcutaneously into 5- to 9-week-old athymic Balb 3T3 *nu/nu* mice. Animals were observed regularly every week up to 12 weeks. Tumour-take incidence and the period required for a tumour to gain the volume of 4180 $mm^3$ were determined. The volume was calculated with the dimensions by considering a tumour as an ellipse.

© 2000 Cancer Research Campaign

### Evaluation of apoptotic phenotype

High molecular weight DNA was extracted from the cells, by the incubation with 0.5% SDS and 50 $\mu$g ml$^{-1}$ proteinase K following the phenol–chlorform extraction. Three micrograms of DNA were electrophoresed in 1.8% agarose gel and stained with ethidium bromide for the observation of ladder formation.

Nucleosome sized DNA fragmentations by apoptosis was also observed by TUNEL assay (Clifton et al, 1998), using Apop Tag (Intergen Co., Oxford, UK) following the manufacturer's instructions. The ratio of positive staining was determined by counting the average of positive nuclei per 100 cells in three independent microscopic fields.

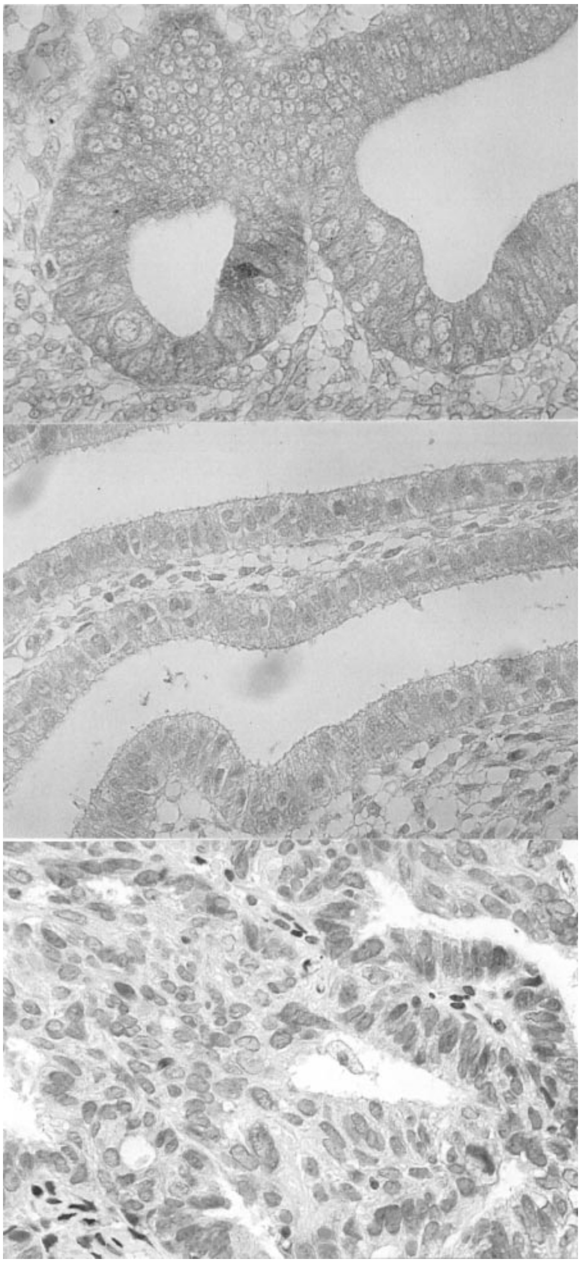

**Figure 1** Immunohistochemical detection of DCC protein in human endometrial glands. Positive staining was observed in human endometrial gland epithelium by anti-DCC antibodies which recognize intracellular (**A**) and extracellular domain (**B**). In contrast, interstitial cells outside the glands in the same photograph and one representative cancer specimens (by antibodies which recognize intracellular domain (**C**)) had no signal. Though all the samples are not shown in this figure, seven out of 12 cancer specimens lost the staining with both of the antibodies

### RESULTS

### Abundant expression of DCC protein in normal human endometrium

First of all, we examined the expression of DCC protein and mRNAs in six human endometrial tissue samples. Immunohistochemical analyses showed the positive staining in normal human endometrium, that was consistent with membrane localization of endometrial gland cells. We used two antibodies, which recognized extracellular (15031A) and intracellular domains (15401A) (Figure 1). Both antibodies showed a similar staining pattern. In turn, we failed to detect positive signals in seven out of twelve endometrial cancer samples (Figure 1), being consistent with our previous observation that half of 28 surgically resected tumours lost *DCC* gene expression (Gima et al, 1994). Immunoblot studies revealed a definite signal band that was an apparent molecular weight 190 kDa in normal human endometrial tissues (Figure 2A). To characterize the DCC protein expressed in human endometrium,

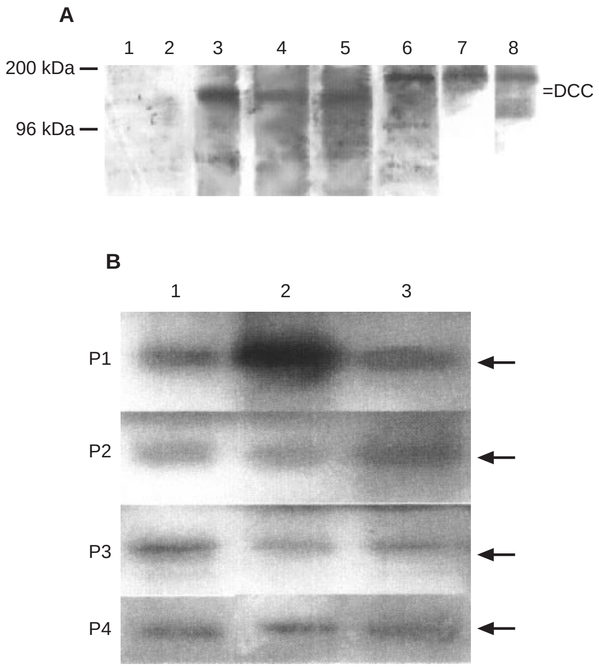

**Figure 2** (**A**) Immunoblot detection of DCC protein in normal endometrium and Ishikawa endometrial carcinoma clones. One hundred µg of protein per lane was analysed by SDS-PAGE and followed by ECL detection with anti-DCC monoclonal antibody Ab-1. Parental Ishikawa, 1; Ishikawa transfected with control plasmid pCMVneo, 2; DCC cDNA transfected Ishikawa clone 55, 3; clone 519, 4; clone 528, 5; independent normal endometrial tissue samples, 6–8. The 190 kDa band in Ishikawa clones and the 175 kDa band in the normal endometrium were detected. (**B**) RT-PCR of RNAs from normal endometrium for DCC coding region. PCR of pDCCCMV-S (1), and RT-PCR of RNAs from clone 55 (2) and normal endometrium (3). Primer sets 1 (P1) covers nt 1 to 1248. Similarly, P2 covers nt 1220 to 2450, P3 covers nt 2403 to 3600 and P4 covers nt 3560 to 4468 respectively. Arrows indicate corresponding PCR products. All the products amplified with the same primer sets showed identical size

*© 2000 Cancer Research Campaign*

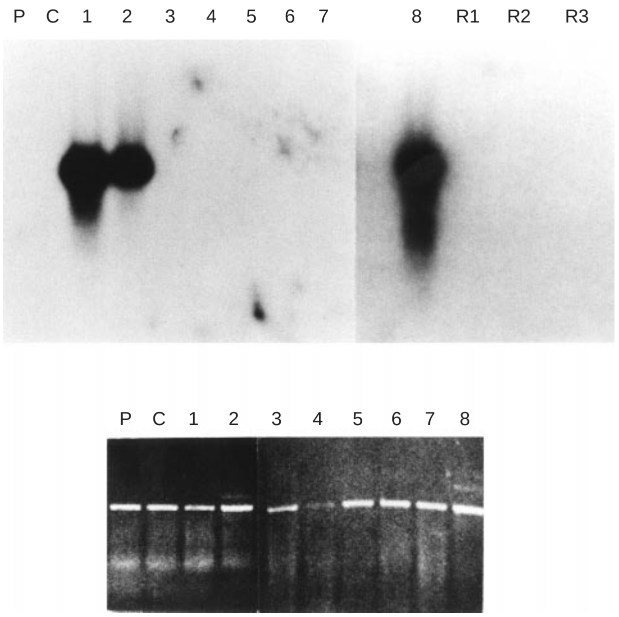

**Figure 3** Transfected DCC transcripts detected by RT-PCR in HHUA clones. RT-PCR with different source of RNA was done by the set of primer (primer 1) spanning nt 1–1249 of DCC cDNA coding region. PCR products were electrophoresed in 1.2% of agarose gel and subjected to hybridization with $^{32}$P-labelled DCC cDNA probe. Parental HHUA, P; HHUA transfected with control plasmid pCMVneo, C; DCC CDNA transfectant clone 7, 1; clone 9, 2; tumours from clone 7, 3–6; tumours from clone 9, 7; clone 11, 8; PCR products with RNA samples without using reverse transcriptase from clone 7, R1; clone 9, R2; clone 11, R3. Only the original pDCCCMV-S transfected clones showed the positive signal, but the tumours generated from the clones did not show the signal. The same results were obtained with other primer pairs (primer 2–4) (data not shown). Amplification of GAPDH of corresponding RNA was also shown below as qualitative control an RT-PCR-based approach was used to determine whether the 175 kDa protein resulted from alternative splicing or post translational modification. By using four sets of primers designed for the full-length ORF, PCR amplification of cDNAs obtained from human endometrium produced products that were identical to the size predicted for the DCC cDNA sequences (Figure 2B). Sequencing of the products showed that human endometrium expressed DCC transcript encoded from the full-length ORF.

We investigated the expression of the DCC ligand, netrin-1 in normal human endometrial tissues (both proliferative and secretory phases) and the human endometrial cancer cell lines, HHUA and Ishikawa. Based on the sequence data (NCBI, Entrez Nucleotide Query; http://HYPERLWK; http://www.ncbi.nlm.nih.gov), we designed two primer sets to amplify netrin-1 cDNAs (190–1060 nt and 1021–1800 nt respectively). However, no amplification was observed in normal human endometrium and two endometrial cancer cell lines.

### Isolation of endometrial cancer cell clones expressing exogenous DCC

To obtain functional evidence for DCC as a tumour suppressor gene associated with endometrial cancer development, we transfected human endometrial carcinoma cells with the human DCC cDNA encoding a complete ORF (Hedrick et al, 1994). The human endometrial cancer cell lines, HHUA and Ishikawa, showed highly transformed growth properties and were tumorigenic in nude mice (Oshimura et al, 1990). A previous report demonstrated the absence of DCC expression in these cells by using RT-PCR analyses (Gima et al, 1994). The plasmid containing full-length DCC cDNA between CMVLTR and rabbit $\beta$-globin poly A signal (generous gift from M Sherman and B Vogelstein) was used for the transfection (pDCCCMV-S) (Hedrick et al, 1994). The ORF of DCC cDNA obtained from human brain predicts a protein of approximately 153 000 molecular weight. The pCMVneo that was the vector without the cDNA was also used for the transfection experiment as a control. pDCCCMV-S and pCMVneo were introduced into the two endometrial cancer cell lines with cationic liposome.

Selected HHUA clones were examined for the restored DCC expression. Western blots using protein extracted from parental HHUA cells and pCMVneo transfectants revealed no signal with anti-DCC monoclonal antibody Ab-1. We also assessed 12 clones obtained from pDCCCMV-S transfectants and failed to detect DCC protein expression in any of these clones. However, hybridization of RT-PCR products with a probe corresponding to DCC cDNA produced an apparent signal in these 12 clones but not in the parental cells or pCMVneo clones (Figure 3). We previously determined that the RT-PCR method detected transcription of 1/10 copy of DCC per cell and this could not detect DCC transcription in the parental HHUA cell (Gima et al, 1994). Thus, the restored transcription in pDCCCMV-S transfectants resulted from the expression of exogeneously introduced DCC cDNA. The relative insensitivity of Western blot analyses may account for the failure to detect DCC protein expressed in the pDCCCMV-S transfectants.

**Table 1** Growth properties of DCC transfected clones

| Clone | Generation time (h) | Saturation density ($\times 10^5$ cm$^{-2}$) | AIG (%) | Tumorigenicity in nude mice (latency)[a] | DCC expression RT-PCR | DCC expression WB |
|---|---|---|---|---|---|---|
| HHUA | 43.7 | 6.7 | 11.6 | 5/5 (53.4) | – | – |
| CT | 59.0 | 6.8 | 11.0 | 7/8 (58.1) | – | – |
| CL7 | 49.2 | 3.9 | 12.0 | 4/8 (68.7) | + | – |
| CL9 | 41.7 | 4.0 | 10.2 | 1/6 (119.0) | + | – |
| CL11 | 79.1 | 4.4 | 13.0 | 0/7 | + | – |
| Ishikawa | 25.7 | 3.4 | 8.7 | 4/4 (72.3) | – | – |
| CT | 33.4 | 3.0 | 6.8 | 4/4 (89.7) | – | – |
| CL55 | 32.9 | 2.7 | 0.3 | 0/4 | + | + |
| CL519 | (166.3)[b] | 1.2 | 0 | 0/4 | + | + |
| CL528 | (305.6)[b] | 0.7 | 0 | ND | + | + |

[a]Days for tumour to 4180 mm$^3$. [b]After a month of culture. ND: not determined.

 © 2000 Cancer Research Campaign

In contrast to HHUA cells, pDCCCMV-S transfection resulted in the appearance of clones expressing a 175 kDa protein detected with anti-DCC Ab-1 monoclonal antibody in Ishikawa cells (Figure 2A). The expression levels in these clones were roughly comparable to that in six human endometrial tissue samples. However, the parental Ishikawa cells and pCMV transfectants failed to express detectable proteins. These results were confirmed by RT-PCR and Southern blots in which highly abundant RT-PCR products were detected in all clones from the pDCCCMV-S transfectants, but the product was absent in the parent and its pCMVneo transfectants. The protein size detected in the pDCCCMV-S transfectants derived from Ishikawa cells was smaller than the one expressed in normal endometrium. As described previously, the apparent difference in molecular mass is likely attributable to post-translational modification, perhaps by glycosylation at potential N-linked glycosylation sites in the extracellular domain (Reale et al, 1994). Each of three representative clones from the HHUA- and Ishikawa-derived pDCCCMV-S transfectants were chosen for further analyses.

### Exogenously introduced DCC suppresses transformed cell properties in endometrial carcinoma cells

DCC expression significantly reduced the saturation density in HHUA cells; a 60–70% reduction was detected in pDCCCMV-S transfectants, compared to those in parent or pCMVneo transfectants (Table 1). However, there were no consistent morphological alternations in pDCCCMV-S transfectants of HHUA grown in monolayer cultures. In addition, expression of exogenously introduced DCC in HHUA cells did not affect in vitro cell growth rate or growth in soft agar cultures with exception of one of three pDCCCMV-S transfectants (clone 11); this showed prolonged population doubling. These findings sharply contrasted to the suppression of in vitro growth properties in Ishikawa cells. Expression of exogenous DCC resulted in a prominent prolongation of population doubling and reduction in saturation density in Ishikawa cells, which were not observed in pCMVneo transfectants. Soft-agar cultures of pDCCCMV-S transfectants of Ishikawa also produced no colonies.

Expression of exogenously introduced DCC in Ishikawa cells resulted in alterations in cell morphology and adhesiveness to the plates (Figure 4). The cells were smaller in size and round in shape. The nuclei were condensed and fragmented (data not shown) in the majority of cells that detached from the plates. When high molecular weight DNAs were extracted from pDCCCMV-S transfectants derived from Ishikawa cells and electrophoresed in 2.0% agarose gel, DNA ladders compatible with apoptotic cell death were observed. DNA fragmentation was seen in all clones from Ishikawa derived pDCCCMV-S transfectants (Figure 5). However, no DNA ladder formation was observed in parent Ishikawa cells, their pCMVneo transfectants or HHUA-derived pDCCCMV-S transfectants. In addition, the apoptotic cell death was also evaluated by using TUNEL assay. Positive signals in nuclei, which indicated apoptotic cell death, was observed in 20% of cells in clone 55, 64% of cells in clone 519 and 22% of cells in clone 528, being contrasted with 6% of parental Ishikawa cells.

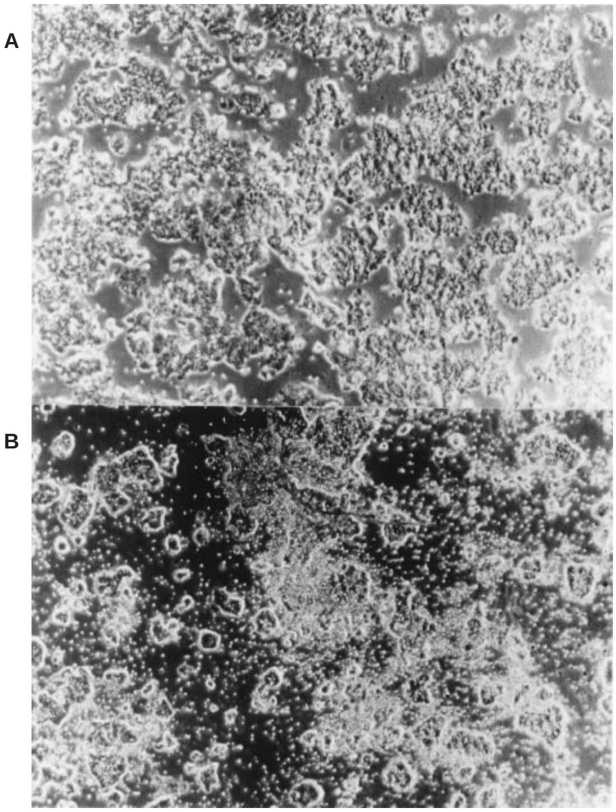

**Figure 4** Morphology of parental Ishikawa cells (**A**) and their pDCCCMV-S transfectant clone 528 (**B**). Most of the clone 528 cells showed the round shape and smaller size, and easily detached from plate surface. Magnification was 100×

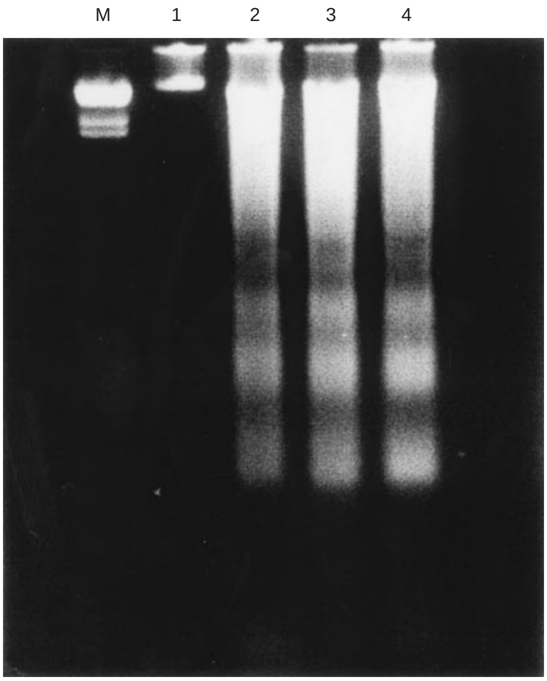

**Figure 5** Apoptotic ladder observed in Ishikawa-derived pDCCCMV-S transfectant clones. Three micrograms of high molecular weight DNA was subjected to each lane. After the electrophoresis in 1.8% agarose gel, the gel was stained by ethidium bromide. Lambda *Hin*dIII marker, M; parental Ishikawa, 1; clone 55, 2; clone 519, 3; clone 528, 4. All Ishikawa-derived pDCCCMV-S transfectant clones which had the apoptotic morphology showed the DNA ladders, that was not seen in parental Ishikawa cells (lane 1)

© 2000 Cancer Research Campaign

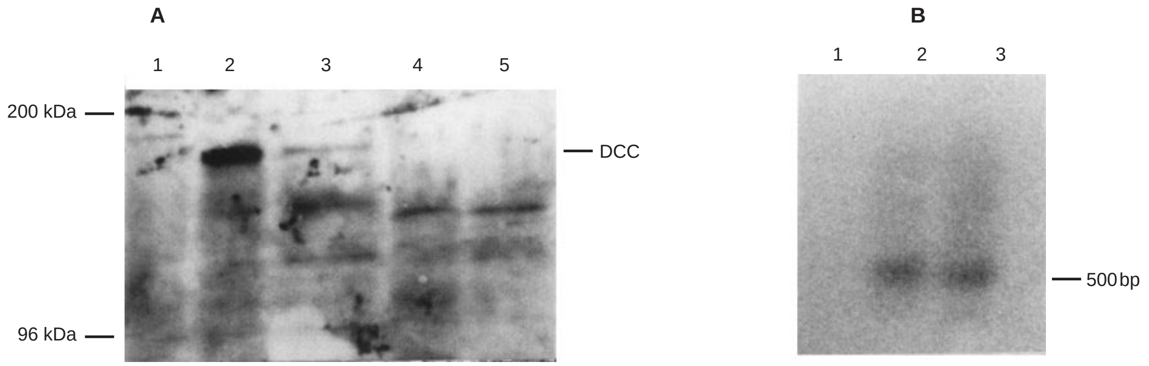

**Figure 6** The alteration of DCC expression during the passage. (**A**) Western blotting of late passaged Ishikawa-derived pDCCCMV transfectant clones. Parental Ishikawa, 1; early passaged clone 55, 2; late passaged (LP) clone 55, 3; LP clone 519, 4; LP clone 528, 5. In the late passaged clones, band intensity was significantly reduced, compared with that in early passaged clone 55. (**B**) RT-PCR with the DCC primer sets were performed and followed by the hybridization with the radiolabelled DCC probe. Parental Ishikawa, 1; LP clone 519, 2; LP clone 528, 3. Although late passaged clone 519 and 528 did not show the signal in Western blot, hybridized signal was observed in these clones

These findings suggest that DCC protein expressed in DCC-deficient Ishikawa endometrial cancer cells induces apoptosis. However, the level of DCC protein expression in HHUA cells may have been too low to induce apoptosis or these cells may respond differently to DCC expression due to other genetic alterations. To address whether low levels of DCC protein expression would affect a potential to induce apoptosis, we isolated a single clone that expressed a reduced level of DCC protein (clone 55-late) and two clones that failed to express DCC protein (clone 519-late and clone 528-late) by prolonged passage of the Ishikawa derived pDCCCMV-S transfectants (Figure 6). RT-PCR-Southern blots were still able to detect DCC cDNA transcription in these late passage clones. The cells did not exhibit DNA fragmentation under condition of either 10% or 0.2% serum. And TUNEL assay positive cells ranged from 4 to 8% in these late passage clones. Thus, the level of DCC protein expression may be critical to induce apoptotic cell death in DCC-deficient endometrial cancer cells.

To determine if DCC suppressed tumorigenicity, we injected pDCCCMV-S transfectants subcutaneously into nude mice (Table 1). The parent HHUA and Ishikawa cells, and pCMVneo transfectants gave rise to rapidly growing tumours. After a latency period of 50–53 days for HHUA and 72–90 days for Ishikawa cells, 20 out of 21 sites injected with either parental cells or their vector transfectants developed progressively growing tumours. In contrast, cells with expression of exogenous DCC exhibited a suppression of tumour incidence and growth. Five tumours developed from 21 sites in which HHUA-derived pDCCCMV-S transfectants were inoculated. Inoculation of Ishikawa-derived pDCCCMV-S transfectants at eight sites produced no tumours. The five tumours produced by the inoculation of HHUA-derived pDCCCMV-S transfectants grew slightly slower than the ones by inoculations of the parent or its pCMVneo transfectants. Average times required to reach 4180 ($mm^3$) in volume ranged from 68 to 119 days. This contrasted with the time for tumours to develop from the parental cell inoculation (53 days) or the pCMVneo transfectant inoculation (58 days). The tumours produced by inoculation of pDCCCMV-S transfectants were poorly differentiated adenocarcinomas similar to those obtained by inoculations of parent or its pCMV transfectants. The cells obtained from these five tumours were examined for DCC mRNA expression, following the incubation in vitro for a few weeks in the presence of G418. DCC mRNA was not detectable by the RT-PCR, indicating deletion or silencing of transfected DCC cDNA sequences (Figure 3).

### DISCUSSION

Transfection of DCC cDNA under control of the strong CMV promoter into two human endometrial carcinoma cells lacking expression of the endogenous *DCC* gene produced cells that expressed different levels of exogenous *DCC*. The transfected HHUA cells expressed a very low level of DCC that was undetectable with Western blot analyses. DCC expression had little effect on in vitro growth of HHUA cells. In contrast, the transfected Ishikawa cells expressed readily detectable levels of DCC protein, which was similar to the levels detected in normal human endometrial tissues. Abundant DCC protein expression in these cells resulted in suppression of transformed cell properties in vitro in terms of doubling time, saturation density and anchorage independent growth. Induction of apoptosis may have contributed to this inhibitory action by DCC. It is likely that the different effects on endometrial carcinoma cells observed in vitro is ascribed to differences in the level of exogenous DCC cDNA expression. Thus, prolonged passage of the Ishikawa-derived pDCCCMV-S transfectants resulted in the appearance of clones in which the DCC expression level was reduced. These late passage clones did not exhibit apoptotic cell death under conditions of either 10% or 0.2% serum. The results suggest that a high level of DCC protein expression that is roughly comparable to that in normal endometrial tissues is disadvantageous for DCC-deficient endometrial cancer cell growth and, as a result, the DCC-expressing cancer cells are eliminated. The DCC cDNA, under control of the CMV promoter, transfected into 1811-NMU-TI cells, which had allelic loss and reduced DCC expression, had little effect on the growth of cells in culture (Klingelhutz et al, 1995). DCC protein was detected in these cells; however, the level can not be compared with that of the cells investigated in the present study.

DCC-negative endometrial carcinoma cells HHUA respond to introduction of DCC cDNA in suppression of tumour formation in vivo, though there was little effect on in vitro cell growth except saturation density. In addition, selective loss of the transcription

© 2000 Cancer Research Campaign was noticed in tumorigenic revertants. Late-passaged Ishikawa clones in which the reconstituted expression of DCC was much lower than early-passaged ones were escaped from apoptosis induction but still kept the suppressed tumorigenic phenotype. It is noteworthy that undetectable levels of DCC protein that has abrogated a potential to suppress in vitro cell growth or induce apoptosis are able to inhibit tumorigenesis of endometrial carcinoma cells. The previously mentioned 1811-NMU-T1 cells transfected with full-length DCC cDNA were non-tumorigenic but still retained the transformed cell properties in vitro. These are compatible with the transfer of a chromosome 18 to endometrial carcinoma cells. *DCC* gene transcription in the microcell hybrid under the control of chromosomal regulatory sequences might be insufficient to achieve the protein levels detected in normal endometrium. Thus, the data implicate the outstanding inhibitory action of DCC protein for tumour formation.

DCC protein is selectively expressed in nerve cells and a small subset of colonic epithelia (Takagi et al, 1996). In addition, we demonstrated here that normal human endometrial cells also expressed high levels of DCC protein. DCC expression is lost during the progression of tumours originated from these three types of cells. In turn, much lower levels of DCC protein is expressed in the normal keratinocytes from which 1181-NMU-T1 cells has been derived (Klingelhutz et al, 1995). A high level of DCC protein expression results in the inhibition of transformed cell properties in vitro in endometrial carcinoma cells whereas little inhibitory effect was shown in 1181-NMU-T1 cells. Thus, it is possible that carcinoma cells originated from tissues that express high levels of DCC protein are more sensitive to exogenous DCC expression.

The present study clearly showed that restoration of DCC protein expression resulted in the induction of apoptotic cell death in DCC-deficient endometrial cancer cells. Human endometrium undergoes morphological and functional alterations in response to cyclic stimulation of ovarian hormones. Gland epithelium converts to decidua-like tissues and results in apoptotic cell death in late secretory phase. DCC induces apoptosis in the absence of ligand binding, but block apoptosis when engaged by netrin-1 (Mehlen et al, 1998). Thus, we amplified the netrin-1 cDNAs from normal endometrial tissues in proliferative and secretory phases to explore the involvement of netrin-1 and DCC-signalling in cyclic transition of endometrium. However, we failed to detect netrin-1 expression in both phases of endometrium. Paracrine secretion of netrin-1 or other ligand binding would play a role to regulate the cyclic transition of endometrium. We also failed to amplify netrin-1 cDNAs from HHUA and Ishikawa endometrial cancer cell lines, suggesting that netrin-1 expression did not show any relationship to the differing in vitro expression levels of the transfected DCC cDNA and its effects.

The functions of DCC associated with tumour suppression remain to be fully elucidated. DCC shares homology with the neural cell adhesion molecules and other cell surface molecules of the immunoglobulin superfamily (Cho et al, 1994) that have been implicated in a wide variety of processes including differentiation and metastasis (Takeichi, 1988; Yun et al, 1996). Preferential localization of DCC protein in a specific subset of differentiated colon mucosal cells suggests its contribution to intestinal differentiation. However, DCC-deficient mice made by transgene technics showed no difference in morphological architecture or in differentiation status of intestinal cells compared to normal littermates. In addition, there was no evidence of increased colon cancer predisposition in the mice in which one DCC allele was knocked out (Fazel et al, 1997). These data may suggest that DCC is not involved in gastrointestinal cell development or carcinogenesis. But transgenic study does not always reflect a native function of a gene in human tissues or involvement of a gene in cancer development. It is generally accepted that carcinogenesis undergoes multistep gene alterations and is influenced by genetic background of individuals. Considering these with the report that colorectal cancers with the absent DCC expression have significantly poor prognosis (Shibata et al, 1996), possible importance of DCC in colorectal carcinogenesis or cellular differentiation is still implied. It should be clarified by further investigations and discussed carefully.

### ACKNOWLEDGEMENTS

We would like to express our appreciation to Drs Michael Sherman and Bert Vogelstein for the generous gift of the DCC expression vector.

### REFERENCES

Brewster SF, Gingell JC, Browne S and Brown KW (1994) Loss of heterozygosity on chromosome 18q is associated with muscle-invasive transitional cell carcinoma of the bladder. *Br J Cancer* **70**: 697–700

Cho KR, Oliner JD, Simons JW, Hedlick L, Fearon ER, Presinger AC, Hedge P, Silvermann GA and Vogelstein B (1994) The DCC gene: structural analysis and mutations in colorectal carcinomas. *Genomics* **19**: 525–531

Chomoczynski P and Sacchi N (1987) Single-step method of RNA isolation by acid guanidium thiocyanate-phenol-chloroform extraction. *Anal Biochem* **162**: 156–159

Clifton DR, Goss RA, Sahni SK, van Antwerp D, Baggs RB, Marder VJ, Silverman DJ and Sporn LA (1998) NF-kappaB-dependent inhibition of apoptosis is essential for host cell survival during *Rickettsia rickettsii* infection. *Proc Natl Acad Sci USA* **95**: 4646–4651

Devileei P, van Vlietl M, Kuipers-Dijkshoorn N, Pearson PL and Cornelisse CJ (1991) Somatic genetic changes on chromosome 18 in breast carcinomas: is the DCC gene involved? *Oncogene* **6**: 311–315

Enomoto T, Fujita M, Cheng C, Nakashima R, Ozaki M, Inoue M and Nomura T (1995) Loss of expression and loss of heterozygosity in the DCC gene in neoplasms of the human female reproductive tract. *Br J Cancer* **71**: 462–467

Fazel A, Dickinson SL, Hermiston ML, Tighe RV, Steen RG, Small CG, Stoeckeli ET, Keino-Masu K, Masu M, Rayburn H, Simons J, Bronson RT, Gordon JI, Tessier-Lavigne M and Weinberg RA (1997) Phenotype of mice lacking functional deleted in colorectal cancer (DCC) gene. *Nature* **386**: 796–804

Fearon ER, Cho KR, Nigro JM, Kern SE, Simons JW, Ruppert JM, Hamilton SR, Preisinger AC, Thomas G, Kinzler KW and Vogelstein B (1990) Identification of a chromosome 18q gene that is altered in colorectal cancers. *Science* **247**: 49–56

Gao X, Honn KV, Grignon D, Sakr W and Chen YQ (1993) Frequent loss of expression and loss of heterozygosity of the putative tumor suppressor gene DCC in prostatic carcinoma. *Cancer Res* **55**: 2723–2727

Gima T, Kato H, Honda T, Imamura T, Sasazuki T and Wake N (1994) *DCC* gene alteration in human endometrial carcinomas. *Int J Cancer* **57**: 480–485

Hedrick L, Cho KR, Fearon ER, Wu TC, Kinzler KW and Vogelstein B (1994) The *DCC* gene product in cellular differentiation and colorectal tumorigenesis. *Genes Dev* **8**: 1174–1183

Hoene MW, Halatsch M-E, Kabl GF and Weinel RJ (1992) Frequent loss of expression of the potential tumor suppressor gene DCC in ductal pancreatic adenocarcinoma. *Cancer Res* **52**: 2616–2619

Imamura T, Arima T, Kato H, Miyamoto S, Sasazuki T and Wake N (1992) Chromosomal deletions and K-*ras* gene mutations in human endometrial carcinomas. *Int J Cancer* **51**: 47–52

Kei J, Zhou TT, Shi YQ, Smolinski KN, Yin J, Souza RF, Appel R, Wang S, Cymes K, Chan O, Abraham JM, Harpaz N and Melzer SJ (1996) Infrequent DPC4 gene mutation in esophageal cancer, gastric cancer and ulcerative colitis-associated neoplasms. *Oncogene* **13**: 2459–2462

© 2000 Cancer Research Campaign 

Keino-Masu K, Masu M, Hinck L, Leonald ED, Chan SS-Y, Culotti JG and Tessier-Lavigne M (1996) Deleted in colorectal cancer (DCC) encodes a netrin receptor. *Cell* **87**: 175–185

Kennedy TE, Serafini T, de la Torre JR and Tessier-Lavigne M (1994) Netrins are diffusible chemotropic factors for commissural axons in the embryonic spinal cord. *Cell* **78**: 425–435

Klingelhutz AJ, Hedrick L, Cho KR and MacDougall JK (1995) The *DCC* gene suppresses the malignant phenotype of transformed human epithelial cells. *Oncogene* **10**: 1581–1586

Mehlen P, Rabizadeh S, Snipeas SJ, Assa-Munt N, Salvesen GS and Bredesen DE (1998) The DCC gene product induces apoptosis by a mechanism requiring receptor proteolysis. *Nature* **395**: 801–804

Miyake K, Inokuchi K, Dan K and Nomura T (1993) Alterations in the deleted in colorectal carcinoma gene in human primary leukemia. *Blood* **82**: 3927–3930

Neuman WI, Wasylyshyn MI, Jacoby R, Erroi E, Angriman I, Montag M, Brasitus T, Michelassi F, Westbrook CA (1991) Evidence for common molecular pathogeneis in colorectal, gastric, and pancreatic cancer. *Genes Chromosomes Cancer* **3**: 468–473

Oshimura M, Kugoh H, Koi M, Shimizu M, Yamada H, Satoh H and Barrett JC (1990) Transfer of normal human chromosome 11 suppresses tumorigenicity in some but not all tumor cell lines. *J Cell Biochem* **42**: 135–142

Reale MA, Hu G, Zafar AI, Getzenberg RH, Levine SM and Fearon ER (1994) Expression and alternative splicing of the deleted in colorectal cancer (*DCC*) gene in normal and malignant tissues. *Cancer Res* **54**: 4439–4501

Ronnett BM, Burks RT, Cho KR and Hedrick L (1997) DCC genetic alterations and expression in endometrial carcinoma. *Mod Pathol* **10**: 38–46

Saegusa M, Hashimura M, Hara A, Okayasu I (1999) Loss of expression of the gene deleted in colon carcinoma (*DCC*) is closely related to histologic differentiation and lymph node metastasis in endometrial carcinoma. *Cancer* **85**: 453–464

Scheek AC and Coons SW (1993) Expression of the tumor suppressor gene DCC in human gliomas. *Cancer Res* **53**: 5605–5609

Serafini T, Colamario SA, Leonald ED, Wang H, Beddington R, Skarnes WC and Tessier-Lavigne M (1996) Netrin-1 is required for commissural axon guidance in the developing vertebrate nervous system. *Cell* **87**: 1001–1014

Shibata D, Reale MA, Lavin P, Silverman M, Fearon ER, Steele G Jr, Jessup JM, Loda M and Summerhayes IA (1996) The DCC protein and prognosis in colorectal cancer. *N Engl J Med* **335**: 1727–1732

Takagi Y, Kohmura H, Futamira M, Kida H, Tanemura H, Shimokawa K and Saji S (1996) Somatic alterations of DPC4 gene in human colorectal cancers in vivo. *Gastroenterology* **111**: 1369–1372

Takeichi M (1988) The cadherins: cell–cell adhesion molecules controlling animal morphogenesis. *Development* **102**: 639–655

Thiagtlingam S, Lengauer C, Leach FS, Schutte M, Hahn SA, Overhauser J, Willson JK, Markovitz S, Hamiltol SR, Kern SE, Kinzler KW and Vogelstein B (1996) Evaluation of candidate tumor suppressor genes on chromosome 18 in colorectal cancers. *Nat Genet* **13**: 343–346

White E (1996) Life, death, and the pursuit of apoptosis. *Genes Dev* **10**: 1–5

Yamada H, Sasaki M, Honda T, Wake N, Boyd J, Oshimura M and Barrett JC (1995) Suppression of endometrial carcinoma cell tumorigenicity by human chromosome 18. *Genes Chromosomes Cancer* **13**: 18–24

Yun Z, Menter DG and Nicolson L (1996) Involvement of integrin alphabeta3 in cell adhesion, motility, and liver metastasis of murine RAWII 7 large cell lymphoma. *Cancer Res* **56**: 3103–3111

 *© 2000 Cancer Research Campaign*
